# Efficacy of cefiderocol in murine models of ventilator-associated pneumonia caused by carbapenem-resistant non-fermenting Gram-negative bacilli, with pharmacokinetic evaluation

**DOI:** 10.1128/spectrum.02568-25

**Published:** 2025-12-23

**Authors:** Kenji Ota, Norihito Kaku, Fujiko Mitsumoto-Kaseida, Naoki Uno, Kosuke Kosai, Takahiro Takazono, Hiroo Hasegawa, Kei Sakamoto, Yoshitomo Morinaga, Koichi Izumikawa, Hiroshi Mukae, Hiroshige Mikamo, Katsunori Yanagihara

**Affiliations:** 1 Department of Laboratory Medicine, Nagasaki University Graduate School of Biomedical Sciences 723477, Nagasaki, Japan; 2 Department of Laboratory Medicine, Nagasaki University Hospital 88380, Nagasaki, Japan; 3 Department of Medical Virology, Nagasaki University Graduate School of Biomedical Sciences 200674 , Nagasaki, Japan; 4 Department of Infectious Diseases, Nagasaki University Graduate School of Biomedical Sciences 200674 , Nagasaki, Japan; 5 Department of Respiratory Medicine, Nagasaki University Graduate School of Biomedical Sciences 723481, Nagasaki, Japan; 6 Department of Microbiology and Immunology, Graduate School of Medicine, Yamaguchi University 13150, Yamaguchi, Japan; 7 Department of Microbiology, Faculty of Medicine, Academic Assembly, University of Toyama 34823, Toyama, Japan; 8 Department of Clinical Infectious Diseases, Aichi Medical University Graduate School of Medicine 12703, Aichi, Japan; The University of Texas at Tyler, Tyler, Texas, USA

**Keywords:** ventilator-associated pneumonia, *Pseudomonas aeruginosa*, *Acinetobacter baumannii*, cefiderocol, pharmacokinetics, mouse model

## Abstract

Ventilator-associated pneumonia (VAP) caused by carbapenem-resistant non-fermenting Gram-negative bacilli poses a serious clinical challenge due to limited treatment options. Cefiderocol (CFDC) is a novel cephalosporin with a catechol moiety that mimics siderophore function, enabling targeted antimicrobial activity against these pathogens. We evaluated the *in vivo* efficacy of CFDC against carbapenem-resistant *Pseudomonas aeruginosa* (CR-Pa) and *Acinetobacter baumannii* (CR-Ab) using murine models of VAP. VAP was induced in neutropenic mice by intratracheal inoculation of bacterial suspension, following endotracheal tube placement to mimic VAP. Pharmacokinetics (PK) analysis was performed to determine CFDC dosing regimens that achieved 70% of the time above the minimum inhibitory concentration (fT > MIC) over 24 h in plasma, which was the average value reported in previous non-clinical studies. In VAP caused by a strain of CR-Ab, CFDC significantly improved the survival rate and reduced bacterial load in the lungs with fT > MIC of 70%. However, in VAP caused by a strain of CR-Pa, CFDC did not significantly improve the survival rate or reduce bacterial load in the lungs, even with fT > MIC of 70%. To evaluate whether a higher fT > MIC of CFDC would enhance *in vivo* efficacy, we assessed the bactericidal effect at higher fT > MIC values. A significant reduction in bacterial load in the lungs was observed in VAP caused by CR-Pa when fT > MIC exceeded 90%. These findings suggest that the optimal CFDC dosing regimen for VAP may differ depending on the causative pathogen, with higher fT > MIC targets potentially necessary for infections caused by *P. aeruginosa*.

**IMPORTANCE:**

Ventilator-associated pneumonia (VAP) caused by drug-resistant non-fermenting bacteria, such as *Pseudomonas aeruginosa* and *Acinetobacter baumannii,* is difficult to treat. Cefiderocol is active against these pathogens, but the amount of drug exposure needed to work in the lung has been uncertain. Using mouse models that mimic VAP, we linked cefiderocol exposure to benefit. *Acinetobacter* infections improved at moderate exposure, whereas *Pseudomonas* infections required much higher exposure to show clear killing in the lungs. These pathogen-specific requirements help explain variable clinical responses and offer actionable targets for selecting doses and schedules. By turning complex pharmacology into simple guidance, our work supports antibiotic stewardship and informs the design of future studies aimed at improving outcomes for patients with difficult-to-treat VAP.

## INTRODUCTION

*Pseudomonas aeruginosa* and *Acinetobacter baumannii* are non-fermenting Gram-negative bacilli, which are major causative pathogens of ventilator-associated pneumonia (VAP) (1, 2). VAP caused by these organisms is associated with high mortality (3, 4), especially when carbapenem resistance is present (4, 5). According to a CDC report, 32,600 cases with 2,700 attributable deaths by multidrug-resistant *P. aeruginosa* and 8,500 cases with 700 attributable deaths by carbapenem-resistant *A. baumannii* (CR-Ab) were estimated in the U.S. in 2017 (6). In addition, the most recent report estimated that carbapenem-resistant *P. aeruginosa* (CR-Pa) and CR-Ab contributed to 57,700 and 38,100 global deaths, respectively, in 2019 (7). The treatment of these pathogens has been challenging due to their rapid development of antimicrobial resistance to multiple classes of antimicrobial agents (8, 9). There are a few available treatment options, such as colistin and tigecycline. However, colistin shows poor lung tissue penetration *in vivo* and has not demonstrated its clinical effectiveness against pneumonia caused by CR-Ab and CR-Pa (10, 11). Tigecycline is not initially effective against *P. aeruginosa*, and clinical evidence supporting its use for pneumonia remains limited (12). Treatment options for these infections therefore remain limited, highlighting the need for new agents.

Cefiderocol (CFDC) is a novel cephalosporin with catechol residue, which mimics a siderophore function (13). Siderophore is a chelating agent against ferric ions, and iron-chelated CFDC is actively transported through the siderophore receptor on the outer membrane of Gram-negative bacteria. CFDC binds to penicillin-binding protein and exerts bactericidal activity. Therefore, its antibacterial activity is exhibited regardless of efflux pump overproduction or porin mutations (14), which consist of the drug-resistant mechanisms in *P. aeruginosa* and *A. baumannii*. Another characteristic of CFDC is improved stability against beta-lactamases, including serine- and metallo-carbapenemases. Previous studies demonstrated *in vitro* efficacy of CFDC against CR-Pa and CR-Ab clinical isolates (15, 16), showing stronger antibacterial effects than ceftazidime, meropenem, levofloxacin, or cefepime. Considering these findings, CFDC has shown potent *in vitro* activity against CR-Pa and CR-Ab and is considered a potential treatment option for VAP caused by CR-Pa and CR-Ab. However, the *in vivo* efficacy of CFDC in VAP caused by CR-Pa and CR-Ab remains unclear.

In this study, we evaluated the efficacy of CFDC against CR-Pa and CR-Ab in the VAP mouse model, defining targeted antibiotic concentrations based on the pharmacodynamic (PD) index associated with β-lactam efficacy, the time above MIC (fT > MIC). To determine the dosing regimens of CFDC, we performed a pharmacokinetics (PK) study using a VAP mouse model caused by CR-Pa and CR-Ab. We subsequently examined whether maintaining this pharmacodynamic target correlates with *in vivo* bactericidal activity.

## MATERIALS AND METHODS

### Study design

This study was designed to evaluate the *in vivo* efficacy of CFDC against CR-Pa and CR-Ab using VAP mouse models. To validate the applicability of the PK/PD-based dosing approach in the VAP mouse models, meropenem (MEM), a well-characterized β-lactam with established PK/PD parameters, was used as a reference compound. The study consisted of three sequential steps. First, a PK study was conducted in the VAP mouse models to determine the appropriate dosing regimens of CFDC and MEM based on targeted fT > MIC values. Second, the applicability of the PK/PD-based dosing regimens was validated using MEM in the same models. Finally, the *in vivo* efficacy of CFDC was assessed in the VAP mouse models.

### VAP mouse models

The VAP mouse models used in this study were previously established and validated in earlier studies (17, 18). CR-Pa, NU3724, was clinically isolated at Nagasaki University Hospital (17), and a CR-Ab isolate harboring a *bla*_oxa-51_-like gene (AMU62852) was kindly provided by Aichi Medical University (18, 19). Specific-pathogen-free (SPF) male ICR mice (6–7 weeks old, 25–30 g body weight) were used for the CR-Pa model, and SPF male ddY mice (6–7 weeks old, 30–35 g body weight) were used for the CR-Ab model. All mice were purchased from Japan SLC, Inc. (Shizuoka, Japan). For both models, mice were treated with cyclophosphamide intraperitoneally at day 4 (150 mg/kg of body weight) and day 1 (100 mg/kg of body weight) before infection (20).

The bacteria were stored at −80°C in a Microbank bead preservation system (Pro-Lab Diagnostics, Ontario, CA) until use.

To prepare the bacterial inoculum, CR-Pa was cultured on Mueller-Hinton II agar (Becton Dickinson, Le Pont de Claix, France) at 37°C overnight. The concentration of CR-Pa was adjusted to 2 × 10^7^ cells/mL in sterile saline, measured using a McFarland turbidimetry (DEN-1B Densitometer, WAKENBTECH, Kyoto, Japan), and appropriately diluted to achieve the target inoculum concentration. CR-Ab was cultured on Mueller-Hinton II agar at 37°C for 18 h and grown in Luria-Bertani broth at 37°C for 6 h. After centrifugation (3,000 × *g*, 15 min), the CR-Ab were resuspended in sterile saline and adjusted to 1 × 10^7^ cells/mL, as estimated by turbidimetry.

Inoculation was performed as described previously (17). Disposable sterile plastic cut-down intravenous catheters with a gauge of 3 Fr (Atom Co., Tokyo, Japan) were cut to a length of 5.0 mm, and a few slits were made at the proximal end to prevent clogging by oral secretions (17). The tube was inserted through the vocal cords into the trachea. Subsequently, bacterial inoculum (0.05 mL; 1 × 10^6^ cells/mL for CR-Pa and 5 × 10^5^ cells/mL for CR-Ab) was inoculated through the outer sheath of the intravenous catheter. Because these two VAP models were independently optimized for each pathogen and mouse strain, the inoculum size differed according to the validated infectious dose required to achieve reproducible infection and mortality in each model (17, 18).

### Pharmacokinetics in VAP mouse models

CFDC was synthesized by Shionogi & Co., Ltd. (Osaka, Japan). MEM and cilastatin were purchased from Wako Pure Chemical Corporation (Osaka, Japan) and Hangzhou APIChem Technology Co., Ltd. (Hangzhou, China), respectively. To determine the appropriate antimicrobial administration dosage, plasma drug concentrations of an infected mouse were measured. The mice infected with CR-Pa were treated with 1 or 10 mg/kg of CFDC or 30 or 300 mg/kg of MEM. The mice infected with CR-Ab were treated with 10 or 100 mg/kg of CFDC or 100 or 500 mg/kg of MEM for CR-Ab. Subsequently, mice were dissected under aseptic conditions to collect the blood via a right ventricular puncture using heparin-coated syringes at each time point (0.083, 0.25, 0.5, 1, 2, 4, and 6 h). Blood was centrifuged for 10 min at 3,000 rpm to separate plasma, which was frozen immediately on dry ice and stored at −20°C prior to analysis. CFDC and MEM concentrations in plasma were determined by the validated liquid chromatography-tandem mass spectrometry method (21). Briefly, mouse plasma samples were protein precipitated using 0.1% trifluoroacetic acid and methanol. The mobile phase A was water/heptafluorobutyric acid, and the mobile phase B was acetonitrile/heptafluorobutyric acid. The internal standard used was S-649266-d12 sodium. For measurement of MEM plasma concentrations, samples were deproteinated with 0.1% formic acid in methanol. The gradients of mobile phases were water/heptafluorobutyric acid and acetonitrile/heptafluorobutyric acid. The lower limit of quantification of CFDC and MEM was 0.500 µg/mL.

### Determination of treatment protocol

We determined the administration dosage of each drug based on the results of PK studies. The MICs for CFDC and MEM were determined using a broth microdilution method in accordance with the guidelines of the CLSI (M100, 29th ed.). According to the guidelines, iron-depleted cation-adjusted Muller-Hinton II broth was used to measure MIC for CFDC. For CFDC, the dilution range was extended below the usual minimum of 0.03 to 0.008 mg/L to enable the accurate determination of pharmacodynamic target attainment (fT > MIC) for PK/PD-based dose design. Although the cefiderocol MIC of the *P. aeruginosa* isolate NU3724 (0.008 mg/L) was lower than typical clinical distributions, this strain was used because only a few carbapenem-resistant *P. aeruginosa* isolates can reproducibly establish a stable VAP model in mice (17).

In designing the treatment protocol, targeted fT > MIC for plasma was set as 70.3% for CFDC, corresponding to the value reported for *P. aeruginosa* in mouse infection models (22). The same target was applied to *A. baumannii* to evaluate whether both pathogens exhibit comparable pharmacodynamic responses at equivalent fT > MIC. In addition, higher exposure groups achieving approximately 90.5% and 100% fT > MIC were included to confirm the dose-dependent relationship between target attainment and bactericidal efficacy. For MEM, the targeted fT > MIC for plasma was set as 30%, based on a previous study showing that 30% fT > MIC achieves bactericidal activity for carbapenems in mouse models (23). These values represented the average PD thresholds observed in non-clinical models and were lower than the clinical target of 100% achieved by the usual regimen for CFDC. Antimicrobial treatment was initiated 3 h post-infection in both VAP groups. In the control group, normal saline was administered to the mice. For further investigation of the dose-dependent bactericidal effect of CFDC, higher concentrations of the drug (to achieve fT > MIC of 90% and 100%) were administered to the infected mice.

### Evaluation of treatment efficacy

In the survival analysis, the treatment was continued up to 120 h post-inoculation, and the survival rates were observed. In bacteriological examinations, the mice were sacrificed at 24 and 48 h post-infection for CR-Pa and CR-Ab VAP models, respectively, corresponding to the time points of maximal bacterial growth and lung injury determined in preliminary validation experiments for each model. For comparison of pharmacodynamic effects across dosing regimens, additional data for *A. baumannii* at 24 h were also evaluated. Subsequently, the lungs were removed and suspended in 1 mL of normal saline and homogenized using a homogenizer (T10 basic ULTRA-TURRAX, Yamato, Fukuoka, Japan). Serial dilutions of the lungs were quantitatively cultured on Mueller-Hinton II agar plates. After 12–16 h incubation, we evaluated the number of visible colonies. The lowest level of detectable counts was 1 × 10^2^ CFU/mL. Bacterial load was expressed as log_10_CFU ± standard errors of the mean (SEM)/lung. In histopathological examinations, whole lungs were collected under aseptic conditions at 24 and 48 h post-infection for CR-Pa and CR-Ab VAP models, respectively. They were fixed in a 10% formalin neutral buffer-methanol solution (Mildform 10NM, Wako, Osaka, Japan), and the lung tissue sections were paraffin-embedded and hematoxylin and eosin stained.

### Statistical analysis

All data were analyzed using Prism version 10.5 (GraphPad Software) and expressed as mean ± SEM. Survival rates were expressed using the Kaplan-Meier method and analyzed using the log-rank test. Differences between the untreated control group and each treatment group were examined using an unpaired t test or Dunnett’s multiple comparison test.

## RESULTS

### Determination of dosing regimens for VAP mouse models

**Fig 1:**
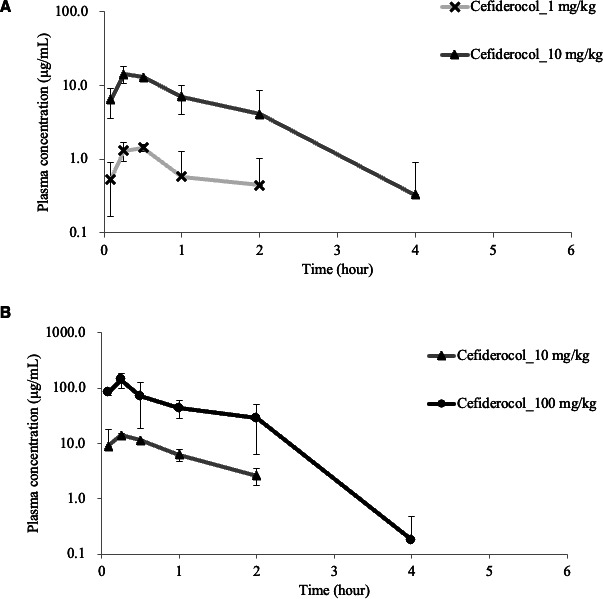
Free drug concentration of CFDC in VAP mouse model. (**A**) In VAP caused by carbapenem-resistant *P. aeruginosa*, 1 and 10 mg/kg of CFDC were intraperitoneally administered at 3 h post-infection. In VAP caused by carbapenem-resistant *Acinetobacter baumannii* (**B**), 10 and 100 mg/kg of CFDC were intraperitoneally administered at 3 h post-infection. In both models, free drug concentration of CFDC in plasma was measured at 0.083, 0.25, 0.5, 1, 2, 4, and 6 h after the administration of CFDC. Because Fig. 1 is plotted on a logarithmic scale, concentrations below the detection limit (zero values) could not be shown in the graph.

The concentration-time profiles of free CFDC and MEM in the plasma of infected mice are shown in Fig. 1 and Fig. S1. PK parameters (Ke, Vc, ka) were calculated using the WinNonlin software (Pharsight, NJ, USA) based on a 1-compartmental model (Table S1). The MICs of CFDC for the CR-Pa and CR-Ab strains were 0.008 and 0.5 mg/L, respectively. The MICs of MEM for CR-Pa and CR-Ab strains were 16 and 128 mg/L, respectively.

Dosing regimens to achieve each targeted fT > MIC were expressed by the unbound fraction of CFDC and MEM. For VAP caused by CR-Pa, CFDC administration of 3 mg/kg every 8 h was employed to achieve an estimated fT > MIC of 76.0%, and for VAP caused by CR-Ab, CFDC administration of 55 mg/kg every 6 h was employed to achieve an estimated fT > MIC of 70.1% (Table 1). For VAP caused by CR-Pa, MEM 110 mg/kg every 8 h was employed to achieve 30%, and for VAP caused by CR-Ab, MEM 1,100 mg/kg every 6 h was employed to achieve 30% (Table S2). These MEM dosing regimens were substantially higher than standard clinical doses due to the elevated MICs of the test strains. The high doses were required to achieve the target fT > MIC values in this model.

**TABLE 1:** Dosing regimens and fT > MIC of CFDC in VAP caused by CR-Pa and CR-Ab^*a*^

VAP mouse models	Dosage (mg/kg)	Interval (h)	fT > MIC (%)
CR-Pa	3	8	76.0
	10	8	90.5
	30	8	100
CR-Ab	55	6	70.1
	210	6	90.5
	390	6	100

^*a*^ Free drug remains above the MIC over 24 h, fT > MIC; VAP, ventilator-associated pneumonia; CR-Pa, carbapenem-resistant *P. aeruginosa*; CFDC, cefiderocol; MEM, meropenem.

### Validation of PK/PD-based dosing using MEM

To validate the appropriateness of the PK/PD-based dosing strategy in the VAP mouse models, MEM was administered at doses designed to achieve the target fT > MIC of approximately 30%, based on previous pharmacodynamic studies in murine infection models. The resulting therapeutic effects were evaluated in terms of survival and bacterial burden in the lungs.

In both CR-Pa and CR-Ab VAP models, MEM treatment led to significant improvements in survival compared to the untreated control groups (Fig. S2A and C). In addition, MEM significantly reduced the bacterial loads in the lungs at 24 h post-infection for CR-Pa (Fig. S2B) and 48 h post-infection for CR-Ab (Fig. S2D). These findings confirm that the PK/PD-guided dosing approach was suitable for evaluating the efficacy of antibiotics in this experimental system.

### Efficacy of CFDC with fT > MIC, 70%

**Fig 2:**
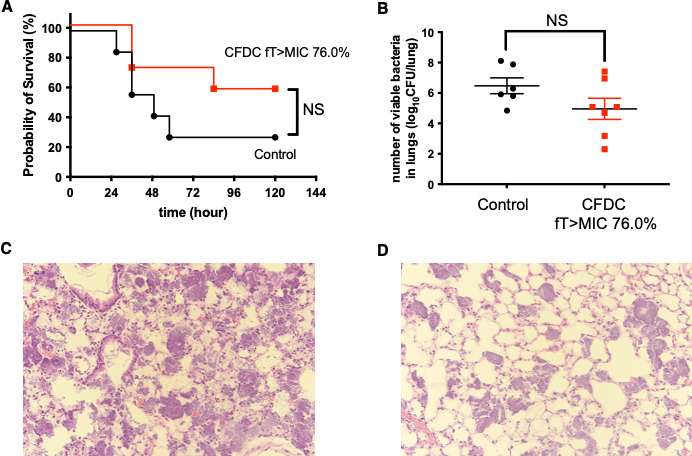
Efficacy of CFDC in VAP caused by CR-Pa. (**A**) Survival rates of mice with VAP caused by CR-Pa were monitored for up to 120 h post-infection. Each group consisted of seven mice. Statistical significance compared to the untreated control group was evaluated using the Kaplan-Meier method with the log-rank test. (**B**) Bacterial loads in the lungs were measured at 24 h post-infection. CFDC treatment was initiated 3 h after infection. The control group consisted of six mice, and the CFDC treatment group consisted of seven mice. (**C and D**) Histopathological examination of the lungs at 24 h post-infection. Hematoxylin and eosin-stained lung sections are shown for untreated mice (**C**) and CFDC-treated mice (**D**), observed at ×40 magnification. NS, not significant.

In VAP caused by CR-Pa, CFDC improved the survival rate, although the difference was not statistically significant compared to the untreated control group (Fig. 2A). CFDC also reduced the bacterial load in the lungs at 24 h post-infection (4.96 ± 0.70 vs 6.47 ± 0.52 in the untreated control group; Fig. 2B), but the difference was not statistically significant. In histopathological evaluation at the same time point, infiltration of inflammatory cells, alveolar hemorrhage, and destruction of alveolar structures were observed in the untreated control lungs of mice with VAP caused by CR-Pa (Fig. 2C). These pathological changes were improved by CFDC (Fig. 2D).

**Fig 3:**
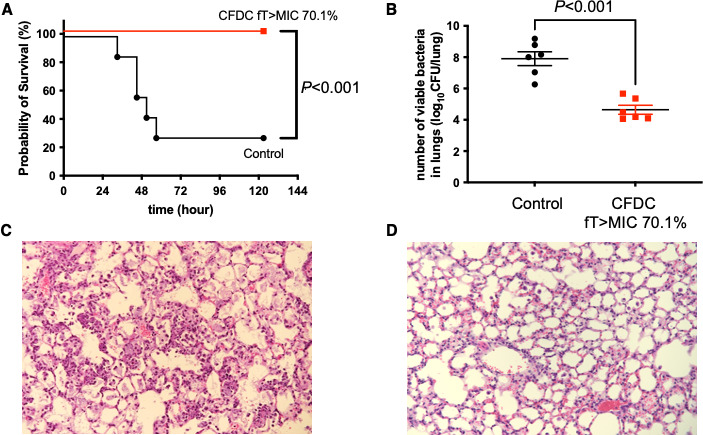
Efficacy of CFDC in VAP caused by CR-Ab. (**A**) Survival rates of mice with VAP caused by CR-Ab were monitored for up to 120 h post-infection. Each group consisted of seven mice. Statistical significance compared to the untreated control group was evaluated using the Kaplan-Meier method with the log-rank test. (**B**) Bacterial loads in the lungs were measured at 48 h post-infection. CFDC treatment was initiated 3 h after infection. Each group consisted of six mice. (**C and D**) Histopathological examination of the lungs at 48 h post-infection. Hematoxylin and eosin-stained lung sections are shown for untreated mice (**C**) and CFDC-treated mice (**D**), observed at ×40 magnification.

In VAP caused by CR-Ab, CFDC significantly improved the survival rate compared to the untreated control group (*P* < 0.001) (Fig. 3A), also significantly decreased the bacterial load in the lungs at 48 h post-infection (4.64 ± 0.29 vs 7.90 ± 0.44, *P* < 0.001) (Fig. 3B). In histopathological evaluation, infiltration of inflammatory cells, alveolar hemorrhage, and destruction of alveolar structures were also observed in the untreated control lungs of mice with VAP caused by CR-Ab (Fig. 3C). These pathological changes were improved by CFDC (Fig. 3D).

### Investigation of dose-dependent bactericidal effect of CFDC

**Fig 4:**
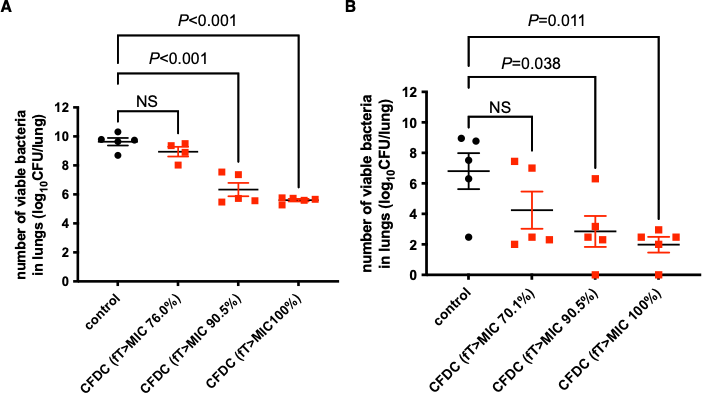
Dose dependency of CFDC in VAP caused by CR-Pa and CR-Ab. Bacterial loads in the lung in VAP caused by CR-Pa (**A**) and CR-Ab (**B**) are shown. In CFDC treatment groups, a target fT > MIC of 70%, 90%, or 100% was set in both models. Treatment was initiated at 3 h post-infection, and bacterial load in the lungs was evaluated at 24 h post-infection. Dunnett’s multiple comparisons test is performed. A *P* value <0.05 is considered statistically significant. Each group consisted of five mice. NS, not significant.

Since CFDC did not improve the survival rate and decrease bacterial load in the lungs significantly in VAP caused by CR-Pa, we assessed the dose-dependent bacterial effect of CFDC. The dose regimens were determined, as shown in Table 1. In both VAP caused by CR-Pa and CR-Ab, CFDC showed a dose-dependent effect on the number of viable bacteria in the lungs. In VAP caused by CR-Pa (Fig. 4A), CFDC significantly decreased the bacterial load in the lungs with fT > MIC 90.0% and fT > MIC 100.0% but not significantly with fT > MIC 70%. Similar results were observed in VAP caused by CR-Ab (Fig. 4B).

## DISCUSSION

We investigated the efficacy of CFDC in VAP caused by carbapenem-resistant pathogens in immunocompromised mice. The treatment of VAP is challenging to clinicians because of the severity, immunodeficiency due to patients’ underlying diseases, and relatively frequent antimicrobial resistance of causative pathogens (24). Since initial treatment failure is one of the risk factors of mortality in VAP (25), appropriate antimicrobial treatment is essential for successful treatment. Nevertheless, limited antimicrobial agents are available to treat VAP caused by multidrug-resistant Gram-negative non-fermenting bacteria, such as *P. aeruginosa* and *A. baumannii*. In addition, the emergence and spread of multidrug-resistant or extensively drug-resistant *P. aeruginosa* and CR-Ab (26, 27) also raise the importance of immediately establishing novel options against multidrug-resistant pathogens.

In the present study, CFDC improved survival rates and decreased the bacterial loads in the lungs in VAP caused by CR-Ab with fT > MIC 70% but not in VAP caused by CR-Pa with it. The MICs of our test strains against CFDC were 0.008 mg/L for CR-Pa and 0.5 mg/L for CR-Ab, both susceptible to CFDC, as most of the clinical isolates in a recent study (16). Using these clinical isolates as possible causative pathogens, we demonstrated the *in vivo* efficacy of CFDC with a clinically available regimen to achieve targeted fT > MIC. Initially, we targeted fT > MIC as 70% for CFDC. The targeted fT > MIC was determined based on the previous study investigating the fT > MIC and bactericidal effect of CFDC in murine thigh and lung infection models (22, 28). Consistent with this finding, the survival improvement and bacterial load reduction were statistically significant for CR-Ab, as expected; however, the bactericidal effect was not confirmed for CR-Pa. Supporting our finding of decreased bactericidal effect, Monogue et al. evaluated the efficacy of CFDC in the neutropenic murine thigh infection model and found that when fT > MIC of CFDC was 80% for strains with MIC of 8 mg/L, the efficacy was proven in only half of the isolates (29). Furthermore, Nakamura et al. reported that the fT > MIC required for bacterial effect showed large variations between individual strains of each bacterial species in mouse thigh and lung infection models (22). Based on these findings, we proceeded to further investigations to evaluate the efficacy of CFDC with higher concentrations and assess its dose dependency. As a result, CFDC significantly decreased the bacterial load in the lungs in CR-Pa and CR-Ab with targeting fT > MIC 90% and 100%. In addition, the dose dependency was observed for both VAP models. These results are concordant with previous studies suggesting that some clinical isolates require higher fT > MIC to show the bactericidal effects of CFDC.

In the clinical setting, high fT > MIC of 100% can be achieved by the usual dosing regimen for CFDC against susceptible pathogens. A previous study demonstrated that fT > MIC of 100% was achieved in humans with the standard dosing regimen of CFDC (2 g administered every 8 h over 3 h infusion) (30). The estimated CFDC concentration achieved by the employed treatment regimen in the present study is much lower than that achieved by the usual human dose. Therefore, our result supports the effectiveness of CFDC in the current clinical dosing regimen. In fact, a previous study evaluating plasma CFDC concentration in humans infected with Gram-negative pathogens, including carbapenem-resistant bacteria, reported that more than 90% probability of target attainment was achieved by clinical dosing regimen against strains with MICs of ≤4 μg/mL (30). In addition, the clinical effectiveness of CDFC against nosocomial pneumonia caused by drug-resistant pathogens has been reported (31, 32). However, in the case series of extensively drug-resistant *A. baumannii* bloodstream infection or VAP patients treated by CFDC (33), failure of microbiological eradication was observed in 54% of patients. The microbiological failure occurred in 80% of patients with inadequate attainment of CFDC determined by trough concentration/MIC, compared with only 29% among those who achieved quasi-optimal or optimal targets. Considering these findings, although our result showed microbial effect under desirable fT > MIC attainment *in vivo*, additional clinical study and careful monitoring are required to treat VAP, especially caused by *A. baumannii*.

In our study, MEM was included as a reference agent to validate the suitability of the PK/PD-based dosing strategy. MEM improved both survival rates and bacterial load reduction in VAP caused by CR-Pa and CR-Ab when administered to achieve a target fT > MIC of over 30%. These results confirmed that our PK/PD-guided approach was appropriate for evaluating *in vivo* efficacy. However, due to the high MICs of the test strains, the MEM dosing regimens used in this study were not clinically feasible and were not intended for therapeutic consideration.

This study had some limitations. First, we evaluated two clinically isolated strains in the VAP mouse model. Therefore, as discussed above, the clinical efficacy of CFDC against *P. aeruginosa* or *A. baumannii* may show variations. In addition, as this study focused on experimentally validating the pharmacodynamic concept underlying CFDC efficacy, the findings should be interpreted as experimental evidence supporting the PK/PD relationship rather than as clinical data to guide infection type-specific or MIC-based dosing adjustments. Furthermore, while *P. aeruginosa* appeared more dose-dependent than *A. baumannii* in this experimental setting, this observation should be interpreted as model-specific pharmacodynamic behavior rather than as evidence of species-specific differences in clinical response. In particular, the CFDC MIC of the *P. aeruginosa* isolate used in this study (0.008 mg/L) was lower than the typical MIC range reported for clinical isolates, and this factor should be considered when interpreting the pharmacodynamic and efficacy results. Our recent epidemiological data from Japan have shown that CFDC MICs for *P. aeruginosa* range from 0.12 to 1 mg/L, and isolates with MICs below 0.03 mg/L are uncommon (34). Thus, while the isolate used in this study represents the lower end of the susceptibility spectrum, its inclusion provides useful insight into pathogen-specific pharmacodynamic behavior, although the generalizability of the findings may be limited. Second, drug concentrations in epithelial lining fluid (ELF) were not measured, and the actual exposed drug concentrations to the infected bacteria were not clarified. In a previous study measuring the plasma and ELF of hospitalized patients with VAP, a parallel correlation between plasma and ELF CFDC concentrations was observed (35). Therefore, we measured plasma concentration, and a dosing regimen was established based on plasma PK analysis. Third, although bacterial inocula were standardized as described in the Materials and Methods, minor variability may have occurred due to the trans-tracheal instillation procedure, which is technically unavoidable in this model. Finally, we did not investigate the inflammatory response by measuring cytokines. Since the purpose of this study is to clarify the bactericidal effect of CFDC and the improvement of survival rate was sufficiently supported by bacterial load reduction, we consider that evaluating the inflammatory response has less importance.

In conclusion, we demonstrated the effectiveness of CFDC against VAP caused by CR-Pa and CR-Ab in an immunocompromised mouse. Since we determined the dose regimens based on the fT > MIC, these data support the indication of CFDC against VAP by drug-resistant Gram-negative pathogens. However, further clinical study is warranted to optimize the fT > MIC for individual bacterial strains.

## Data Availability

The data sets generated and analyzed during the current study are not publicly available due to the involvement of proprietary methods and materials provided by the funding body, but are available from the corresponding author upon reasonable request.

## SUPPLEMENTAL MATERIAL

Fig. S1 spectrum.02568-25-s0001.tiff Free drug concentration of MEM in VAP mouse model.

Fig. S2 spectrum.02568-25-s0002.tiff Efficacy of MEM in VAP caused by CR-Pa and CR-Ab.

Supplemental tables spectrum.02568-25-s0003.docx Tables S1 and S2.

ASM does not own the copyrights to Supplemental Material that may be linked to, or accessed through, an article. The authors have granted ASM a non-exclusive, world-wide license to publish the Supplemental Material files. Please contact the corresponding author directly for reuse.
