# Supplementary figures and images for: Efficacy of cefiderocol in murine models of ventilator-associated pneumonia caused by carbapenem-resistant non-fermenting Gram-negative bacilli, with pharmacokinetic evaluation
Source: Microbiol Spectr. 2025 Dec 23;14(2):e02568-25. doi: 10.1128/spectrum.02568-25 (PMC12889036; doi:10.1128/spectrum.02568-25)

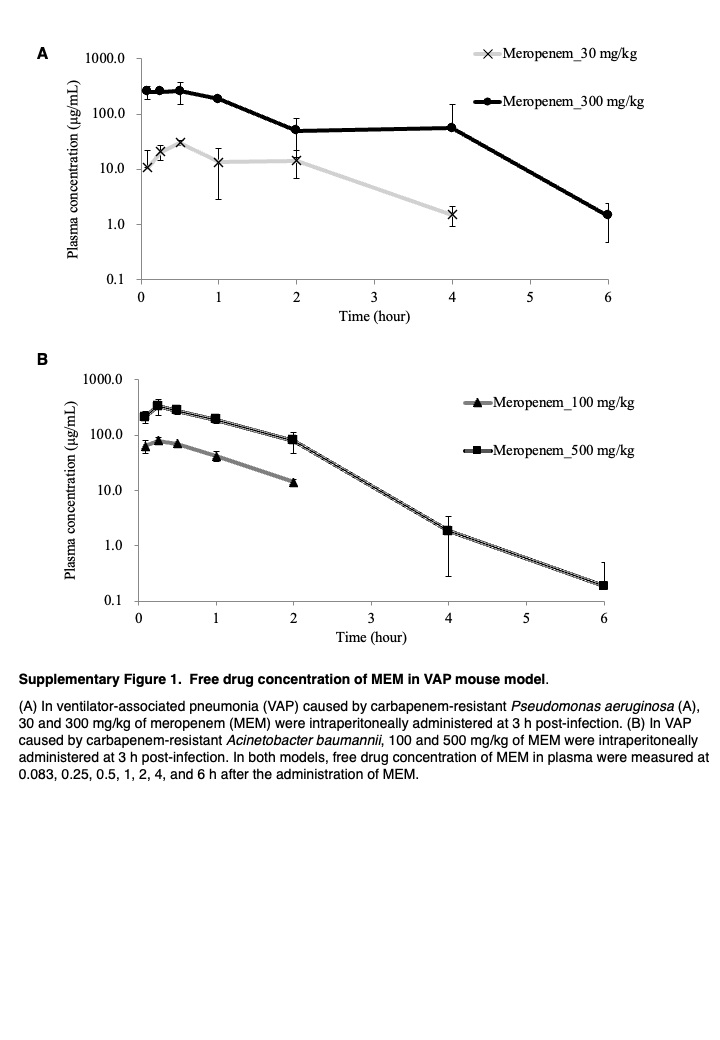

Supplement: Fig. S1 — Free drug concentration of MEM in VAP mouse model. [file spectrum.02568-25-s0001.tiff]

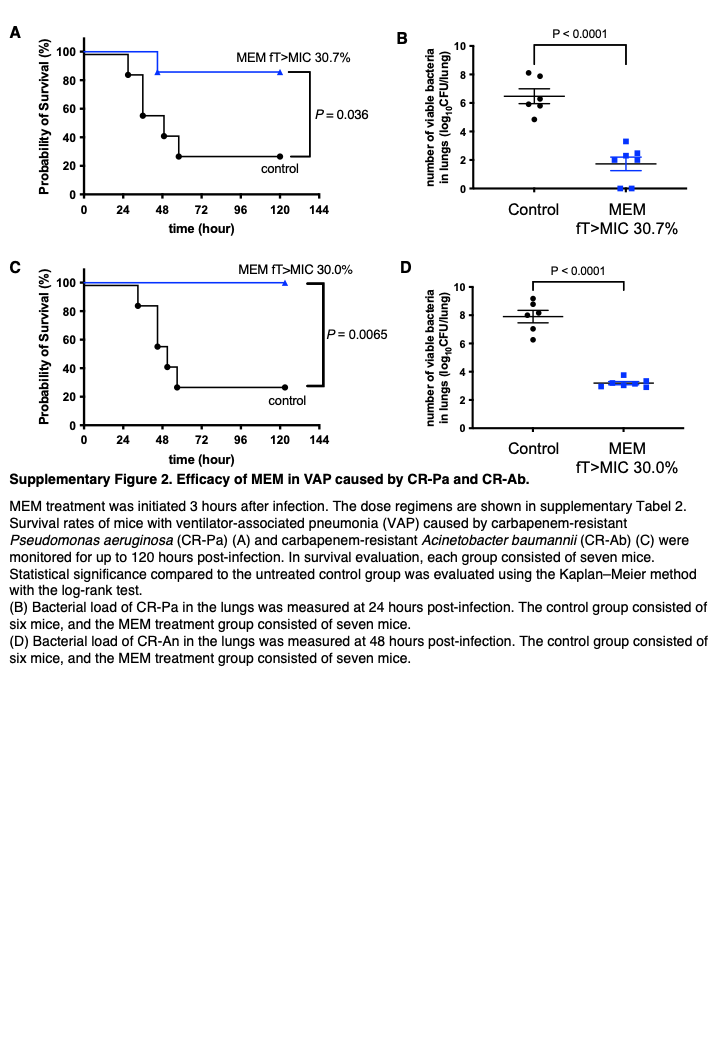

Supplement: Fig. S2 — Efficacy of MEM in VAP caused by CR-Pa and CR-Ab. [file spectrum.02568-25-s0002.tiff]
