# Supplementary material for: Efficacy of cefiderocol in murine models of ventilator-associated pneumonia caused by carbapenem-resistant non-fermenting Gram-negative bacilli, with pharmacokinetic evaluation
Source: Microbiol Spectr. 2025 Dec 23;14(2):e02568-25. doi: 10.1128/spectrum.02568-25 (PMC12889036; doi:10.1128/spectrum.02568-25)
Supplement: Supplemental tables — Tables S1 and S2. [file spectrum.02568-25-s0003.docx]

**Supplementary Table 1**. PK parameters of CFDC and MEM following a single intraperitoneal administration to neutropenic mice infected by CR-Pa and CR-Ab

| Organism | Compound | Dose (mg/kg) | Ke (/hr) | Vc (L/kg) | Ka (/hr) |
| --- | --- | --- | --- | --- | --- |
| CR-Pa | CFDC | 10 mg/kg | 1.033 | 0.514 | 6.283 |
|  | MEM | 300 mg/kg | 0.648 | 0.952 | 20.823 |
| CR-Ab | CFDC | 100 mg/kg | 1.095 | 0.928 | 9.491 |
|  | MEM | 100 mg/kg | 3.429 | 0.146 | 1.960 |

PK parameters were determined using the drug concentrations expressed in Figure 1 and supplementary Figure 1. carbapenem-resistant *Pseudomonas aeruginosa* (CR-Pa); carbapenem-resistant *Acinetobacter baumannii* (CR-Ab); cefiderocol, CFDC; meropenem, MEM.

**Supplementary Table 2.** Dosing regimens and fT>MIC of MEM in VAP caused by CR-Pa and CR-Ab

| VAP Mouse models | dosage | interval | fT>MIC |
| --- | --- | --- | --- |
| CR-Pa | 110 mg/kg | 8 h | 30.7 % |
| CR-Ab | 1,100 mg/kg | 6 h | 30.0% |
| Free drug remains above the MIC over 24 h, fT>MIC; ventilator-associated pneumonia, VAP; carbapenem-resistant *Pseudomonas aeruginosa* (CR-Pa); meropenem, MEM. MEM was administered with the same amount of cilastatin. For MEM, the targeted fT>MIC for plasma was set at 30%, based on previous studies showing that 30% fT>MIC achieves bactericidal activity for carbapenems in mouse models. | | | |
